# A Novel Approach to Identifying Barriers and Facilitators in Raising a Child With Type 1 Diabetes: Qualitative Analysis of Caregiver Blogs

**DOI:** 10.2196/diabetes.8966

**Published:** 2017-10-26

**Authors:** Tamara K Oser, Sean M Oser, Erin L McGinley, Heather L Stuckey

**Affiliations:** 1 Department of Family and Community Medicine Penn State College of Medicine Hershey, PA United States; 2 Departments of Internal Medicine, Humanities, and Public Health Sciences Penn State College of Medicine Hershey, PA United States

**Keywords:** type 1 diabetes, blogs, caregiver, self-management, social media, peer support, Internet

## Abstract

**Background:**

With rising incidence of type 1 diabetes (T1D) diagnoses among children and the high levels of distress experienced by the caregivers of these children, caregiver support is becoming increasingly important. Historically, relatively few support resources have existed. Increasing use of the Internet, and blogs in particular, has seen a growth of peer support between caregivers of children with T1D. However, little is known about the type and quality of information shared on T1D caregiver blogs. At the same time, the information on such blogs offers a new window into what challenges and successes caregivers experience in helping to manage their children’s T1D.

**Objective:**

The purpose of this study was to (1) analyze blogs of caregivers to children with T1D to better understand the challenges and successes they face in raising a child with T1D, and (2) assess the blogs for the presence of unsafe or inaccurate clinical information or advice.

**Methods:**

An inductive thematic qualitative study was conducted of three blogs authored by caregivers of children living with T1D, which included 140 unique blog posts and 663 associated comments. Two physician investigators evaluated the blogs for presence of clinical or medical misinformation.

**Results:**

Five major themes emerged: (1) the impact of the child’s diagnosis, (2) the burden of intense self-management experienced in caring for a child with T1D, (3) caregivers’ use of technology to ease their fear of hypoglycemia and impacts that device alarms associated with this technology have on caregiver burden, (4) caregivers’ perceptions of frequently missed or delayed diagnosis of T1D and the frustration this causes, and (5) the resilience that caregivers develop despite the burdens they experience. Misinformation was exceedingly rare and benign when it did occur.

**Conclusions:**

Blog analysis represents a novel approach to understand the T1D caregiver’s experience. This qualitative study found many challenges that caregivers face in raising a child with T1D. Despite the many barriers caregivers face in managing their children’s T1D, they find support through advocacy efforts and peer-to-peer blogging. Blogs provide a unique avenue for support, with only rare and benign findings of medical misinformation, and may be a resource that diabetes care providers can consider offering to families for support.

## Introduction

Type 1 diabetes (T1D) is an increasingly common chronic condition among children, with the incidence growing 3% to 5% per year since 1960, and with more rapid growth since 1990 [1]. Between 2001 and 2009, there was a 21% increase in the prevalence of T1D in people younger than 20 years [2]. Globally, 78,000 children ages 14 years and younger are diagnosed every year [3]. Having a child diagnosed with a chronic and life-threatening illness such as T1D is highly distressing to the caregiver and can lead to parental depression, acute stress, or posttraumatic stress reactions [4-7]. Higher levels of acute distress in parents predict not only parents’ development of persistent mental health difficulties [8,9], but also longer-term psychological, behavioral, and general well-being outcomes among their children [10-13]. Among youth with T1D, high levels of parental distress have been associated with poorer health outcomes in both the children and the parents [14-16]. In their recent position statement regarding psychosocial care for people with diabetes, the American Diabetes Association recommended that providers include caregivers in their assessment of diabetes distress, depression, and anxiety (level B recommendation) [17].

In addition to psychosocial stressors, the burden of T1D management often falls to the child’s caregiver(s), and few conditions require as much self-management as T1D. Although it is generally recommended that patients with T1D meet with their diabetes health care team quarterly, this results in but a few hours of in-person contact, leaving more than 8000 hours per year that T1D must be self-managed [18]. Caregivers to children with T1D often describe feeling isolated in managing the complex, relentless demands of this disease [19]. They face numerous challenges in adjusting to a T1D diagnosis and in managing their child’s T1D care.

It is therefore important to understand the specific challenges that caregivers face and help identify novel sources of support. Despite advances in technology for the diabetes community, health care providers (HCPs) have relatively few resources to which they may refer caregivers for support. There are even fewer that are easily and rapidly accessible. Although there are examples of early evidence supporting online and mobile resources [20-23], there is comparatively less known about already widespread and publicly available resources outside of the research environment. Blogs—public online journals—have become popular in the age of social media. Blogs represent a growing online resource for caregivers to children with T1D to initiate and receive support. Many bloggers feel that by sharing their struggles and celebrating their successes, blogs help them to more effectively approach the daily self-management T1D requires [18]. A referral from a HCP to high-quality blogs might serve as a valuable tool in lessening the psychosocial burden caregivers of children with T1D carry with them, but to date there has been little data on the types or quality of information present on health care-related blogs. In addition, HCPs may be hesitant to refer caregivers to blogs for support given the lack of data on medical misinformation that may exist on blogs.

The objectives of this study were to better understand the issues faced by caregivers to children with T1D via qualitative analysis of blog content and to assess the types and quality of information found on blogs, specifically focused on identifying potential misinformation.

## Methods

### Sample Description and Recruitment

Three publicly available online blogs authored by caregivers to children living with T1D were analyzed to identify barriers and facilitators in their experiences caring for their children with T1D. This study was approved by the Penn State College of Medicine Institutional Review Board (STUDY00000870). Blogs were selected using a strategy described by D’Auria [24], based on a Google search for “parent blog diabetes,” and validated by concurrence with the blogs’ inclusion as a top blog for parents of children with diabetes. Bloggers were recruited via email and consented to allow retrospective analysis of publicly available blog entries and comments posted on their sites from June 1, 2012 to August 31, 2014. Blog posts, including comments, were imported into NVivo 10 (QSR International) [25].

### Inductive Thematic Analysis

After reviewing the blogs and noting initial impressions, a codebook was developed and revised through ongoing discussions among the study team. To first establish Cohen kappa [26], the primary coders (EM, TO) each coded 10% of the dataset. Initial kappa was .920 and a subsequent recheck after additional coding yielded kappa .934. With high interrater reliability established through kappa as well as through group discussion, the primary coders then coded the remaining blog posts and comments individually. Project team meetings included biweekly coding audits. For any discrepancies found, the project team discussed the current meaning of the code and either further modified the code to reflect the correct meaning or revised the coding based on group consensus.

Coding proceeded until saturation, which was determined through a combination of three indicators: (1) use of a saturation table [27], (2) finding that no further edits to the codebook were necessary any longer (and then continuing to code an additional 10% of the sample to corroborate this), and (3) when the study team felt confident that no new themes were being uncovered.

The research team employed inductive thematic analysis [28] to construct emergent themes. The project team reviewed the dataset in multiple ways. For the codes most frequently used, the associated content was reviewed to identify emergent themes. A coding matrix was also produced to identify highly coincident codes and the content associated with them (content frequently coded simultaneously to each of two codes), and to further explore the thematic relationships between them. All members of the study team agreed on the emergent themes presented.

### Clinical Review of Blog Content for Medical Misinformation

Two physicians (TO, SO) reviewed the entire dataset to identify any occurrences of information that was clinically inaccurate, incorrect, or misleading, or content that might be construed as medical advice.

## Results

### Study Sample

Three online blogs were analyzed, representing 140 blog posts and 663 associated comments. All were publicly available on the Internet without registration or permission. Characteristics of the dataset are presented in Table 1. Two blogs were authored by mothers and one by a father. Represented families include both single-parent and two-parent families, as well as families with one, two, and three children with T1D. During the period of reviewed blog posts, the bloggers’ children with diabetes ranged in age from 4 to 16 years.

**Table 1:** Participant blog characteristics.

Blog name	Caregiver role	Family characteristics	Age of children when diagnosed with T1D (gender)	Age of children at time of blog posts	Blog posts during study period	Associated comments
Candy Hearts [29]	Mother	Two-parent family	2 years (female)	9-11 years	43	164
Bleeding Finger Blog [30]	Father	Two-parent family	3 years (female)	6-8 years	47	117
			3.5 years (female)	4-6 years		
Our Diabetic Life [31]	Mother	Single-parent family	8 months (male)	14-16 years	50	382
			2.5 years (male)	8-10 years		
			5.5 years (male)	10-12 years		
Totals					140	663

### Emergent Themes From Inductive Thematic Analysis

Qualitative analysis of blogs yielded five major themes about the caregivers’ experiences of caring for their children with T1D.

#### Theme 1: Fear and Worry Are Common, Starting at the Time of Diagnosis, and Although the Fears and Worries Change in Ensuing Years, These Emotions Persist

Caregivers’ blog posts described the worry they felt at the time of the diagnosis when they wondered how they would care for their newly diagnosed child:

But one day you were not well. Worry set in. We watched you in fear in the hospital bed.

All the while, I sat there, wondering how I would be able to keep her alive without a team of nurses and doctors waiting just outside the door.

Beyond the diagnosis period, fear and worry persist. There is a particular fear of hypoglycemia:

How do I accurately describe the worry that lays wait in my stomach when a child announces a very low number, the choking responsibility of life, and the rolodex of emergency protocols that run through my brain?

I want you to know, when there is an extreme low, and your child is sitting with a blank look in front of you, barely able to speak...I have been there. I have felt the confusion, the panic, and the deep worry you have in your heart.

Parents described in great detail the fear they have that their children will die of hypoglycemia, particularly at night while the child is sleeping. They refer to the emotions they face as they walk to check on their child in the middle of the night or the next morning (“the walk that never ends, the walk to check for disaster”) and the feelings they experience wondering if their child is still alive (“the feeling in my throat as I lean against a bedroom door jamb, waiting for their chests to rise and fall in the morning is a terrible feeling”).

#### Theme 2: Caregivers Experience Unrelenting Physical and Emotional Burdens Related to the Intense Management Demands of Caring for a Child with T1D

Caregivers are challenged by the heavy burden that diabetes brings to the family unit, including the sheer work involved in managing a condition that has a 24/7/365 presence. A commonly expressed pressure is the burden of “having to know what their blood sugars are” at all times. The constant attention to detail causes caregivers to become “worn down.” Caregivers discussed the unrelenting presence T1D has in their lives:

I got up early to give her a breakfast bolus 2 hours before the start of testing with a solid 20-minute prebolus in an attempt to slow/minimize/prevent an astronomical breakfast spike with cognitive function.

How can I lament about the laboriousness of this disease, the constant stream of numbers knocking, knocking, knocking all the livelong day, and the infuriating knowledge that there will never ever, ever be a break from this, when we can take walks by the ocean as a family?

How do I explain to you that some nights the exhaustion holds me like a straight jacket...that the nights are all encompassing, and I will my tears to fall back into my body rather than intentionally give in to the fear and exhaustion?

Despite these efforts, diabetes can be unpredictable. Even stringent efforts to follow treatment parameters result in frequent glucose levels outside the target range: “I think one of the hardest parts is the unpredictability and no rhyme or reason—it makes it impossible to understand and comprehend, which then makes it frustrating when fixes don’t [work].” This unpredictability only adds to caregivers’ self-described burden of lost sleep: “While friends were boasting about weekend getaways and trying new restaurants, we were holding our breath to see if the pizza bolus from dinner would wreak havoc on a good night’s sleep.” They vividly described attempting to control their children’s blood glucose levels at night to keep them safe: “I tested her blood sugar every 2 hours between 10pm and 6am in an attempt to catch any fluctuations that might require an intervention…” and “I know that there were many nights she lost hours and hours of sleep to make sure the kids were safe for me.” This leads to frustration (“I want you to know, when your alarm goes off in the middle of the night and you want to throw your alarm clock out the window...I have been there”) and the desire for a good night’s sleep (“Kind of weird, a full night sleep for a birthday present, a half night with 2 hours of REM will do. A good night sleep, it has been a while, whatever a good night of sleep is”).

Related to lost sleep and the desire to preserve some sleep, caregivers discussed the struggle to balance the children’s glucose control with their own need for sleep, and the feeling that one must be sacrificed for the other (“Should I treat gently and wake in a couple hours to see where they are going...or...should I treat in such a way that I know they will be safe...so I can sleep”) and their discomfort with this decision (“Higher numbers for my boys to ensure sleep for myself is sometimes a necessary trade off, but never a comfortable one”).

Finally, caregivers discussed the persistently bothersome memories of the time around diagnosis (“Yes, I am aware it’s been 9 years since her diagnosis. I still cry when I talk about it”) and how the diagnosis changed life so dramatically for the family (“Life changed, abruptly, never returning close to what it was before”).

#### Theme 3: Caregivers Use Technology to Help With Self-Management and the Fear of Hypoglycemia, and Such Technology Is Generally Seen As Quite Helpful, but Device Alarms Can Also Be Intrusive and Add to the Burden Felt by Caregivers

Caregivers described their excitement over new devices (“I realize an insulin pump may not be the most exciting toy for most of the world, but it’s big stuff in this house—and many other homes, too”) and how they help in decreasing the self-management burden of T1D:

It’s kind of odd, getting excited about a medical device, but it makes a crappy disease a little easier.

...both my daughters switched to the same insulin pump...and [continuous glucose monitor (CGM)] this last summer. This has made diabetes management, for us, a lot easier. I’ve publicly endorsed the [CGM] (some quality issues are there however, like buttons falling off, power port cover coming off) but it is a great tool. I love the range, it catches our daughters upstairs when they are playing or sleeping; the accuracy I find is great.

At the same time, the device alarms were noted as intrusive: “Somewhere, a CGM alarmed, and into the story enters diabetes as the main antagonist” and “It beeps all the time.”

#### Theme 4: Many Caregivers Are Especially Bothered by What They Perceive to Be the Frequently Missed and/or Delayed Diagnosis of T1D

There was an unusually large outpouring of comments by caregivers who recalled their child’s diagnosis as initially missed and/or substantially delayed. This most frequently comes to light when news is spread online about another child recently diagnosed quite late or even after dying: “The symptoms, the physician responses, the results, and the outrage at how something could have been so easily caught, diagnosed and treated without taking our children all the way to death’s door.” Some caregivers recall being told that their ill child had “a virus” or “the flu” without testing being done, days or even weeks before serum or urine glucose testing was eventually ordered and led to the diagnosis of T1D. There were also strong feelings among T1D caregivers that in children less fortunate than theirs, who were only diagnosed in late (and quickly fatal) ketoacidosis, their deaths could have been prevented if the children had received glucose testing earlier in their illness. They expressed concern that “many medical professionals just don’t understand how quickly [diabetic ketoacidosis] can turn life-threatening.”

#### Theme 5: Despite the Fears and Frustrations That Caregivers Experience, They Demonstrate Resilience, Often Through Advocacy Efforts and Peer Support Through Blogs

Quite often, caregiver resilience takes the form of advocacy efforts, as there is substantial discussion in blogs of caregivers’ efforts to promote public awareness, to become involved in advocacy organizations, and to encourage others to do the same. Caregivers encourage one another to “change the world” through “sharing their stories” and inspire each other to “cure this thing!” They discuss the need to make their voices heard in political, industrial, and community venues: “Think about what you want your lawmakers to know about living with type 1 diabetes” and “There are policy makers, pharma companies, news outlets, and simply neighbors in our immediate area that need to hear our collective voices.” They advocate on behalf of their children, who they feel are often too young to do so themselves: “I want to make my voice heard and speak for my daughter until she can learn to speak for herself.”

Support for caregivers is discussed frequently. Blogging is used to provide support to peers, to receive support from peers, and as a mechanism for processing and coping. Those who found blogs at the time of their child’s diagnosis described what an important and highly valued source of support blogs can be: “I found your blog early in our journey and it gave me so much more than you will ever know” and “Honestly I do not know what I would have done had I not found your blog.” Bloggers encouraged others to blog: “Only you can tell your story, and that story might be the one that connects with someone and makes a difference in their life.” They also discussed the importance of the peer-to-peer support received through blogs: “Knowing that there are others out there going through the same things helps other people so much.”

### Medical Advice/Misinformation

Two physician members of the study team (TO, SO) reviewed all posts and comments during the study period to identify instances of bloggers or commenters providing information that was clinically incorrect or inaccurate, or that could be perceived as medical advice. No instances of medical misinformation were found among the 140 blog posts reviewed. In the 663 comments associated with those blog posts, two instances of possible medical misinformation or medical advice were found:

I have read about cats who were very good at giving alerts when a diabetic member of the family had a low blood sugar. Daisy may become very good at it too, if you give her a reward each time she does it.

This comment was considered to be medical misinformation because it could be interpreted by a blog visitor as encouragement to try to train household pets to detect hypoglycemia, a practice that is not recommended or supported by the literature.

His blood sugar was 129... a bit higher than I would’ve ordinarily liked, but considering he was sick and had just drank some apple juice helps explain it (as did a lot of [diabetes online community] reassurance).

This comment was considered to be medical misinformation because the commenter relied on his/her own understanding and reassurance from lay users online, rather than consulting with a HCP to interpret a glucose measurement in their child without diabetes (not their child with diabetes). This raised concern that the comment might encourage others to do the same.

Conversely, all blogs analyzed contained a general statement/disclaimer instructing readers that the blogs’ content should not be considered medical advice and encouraging them to consult a HCP for any medical information. For example, the blogger from Our Diabetic Life provides the following statement:

I can guesstimate a bolus in lightning speed. I can check my boys’ blood sugars in the wee hours in the morning, half-asleep, with only one eye open. I can do a lot of things...but one thing I can’t do is be your child’s endocrinologist. Everything on this blog works for our family, but might not work for yours. Funny thing diabetes, one size does not fit all. If you see some technique here that you would like to try, call your doctor, use common sense, and remember: I am not a doctor...I’m just a mother of three boys with type 1 diabetes. That is it. Mother. Not doctor. Blogger. Not doctor. Friend. Not doctor...

## Discussion

### Principal Results

Blogs tell a story. They allow narrative expression of an individual’s experience, which can have significant health benefits [32,33]. They allow insight into the personal, day-to-day issues faced by families living with T1D. They allow us to see their struggles and challenges as well as their successes. They also allow us to witness the interactions of peers as they provide support to one another.

Using this novel approach of blog analysis, we found that caregivers of children with T1D experience many challenges, starting from the time surrounding their child’s diagnosis and continuing forward. We found that caregivers experience fear and worry at the time of their child’s diagnosis, and that this fear persists, specifically surrounding hypoglycemia, especially at night. This finding is consistent with other studies of T1D caregivers conducted through more traditional means [34-37], but to our knowledge, this is the first study to specifically focus on public blog analysis as a primary means to examine such issues. Also consistent with research by Lowes et al [38,39], we found that there is some degree of chronic sorrow, with caregivers continuing to talk about their child’s diagnosis on blogs, even years later. Caregivers also discuss the frustration and anger that arise from their perception that there is too often a delay or misdiagnosis surrounding the initial presentation of T1D in children.

Interestingly, there was very little discussion of glycated hemoglobin A_1c_ in the caregiver blogs studied, although glycated hemoglobin A_1c_ is often a focus of most HCPs in evaluating the overall quality of diabetes management in individuals with T1D. During blog discussions, caregivers focus more on immediate management issues, such as preventing hypoglycemia at night. Caregivers often discuss fear of nighttime hypoglycemia and even death due to nighttime hypoglycemia, but there is not much discussion about long-term diabetes complications. There are few studies focused on providing behavioral interventions to children with T1D and their parents to reduce fear of hypoglycemia [40-42], and our study supports the need for future research to target pediatric interventions to address parents’ fear of hypoglycemia, perhaps through education and support.

Caregivers assume significant emotional and physical burdens in caring for their children with T1D. Although these burdens are numerous and varied, they are exemplified by prominent online discussions of lost sleep due to the unpredictability of diabetes, which is further compounded by alarms from diabetes management devices. Such devices are certainly considered helpful, but they can also be seen as intrusive as other studies have also shown [43,44].

Despite this, caregivers are resilient. They find support from one another through blogs, and they encourage each other to advocate for change regarding issues they find burdensome, including public misunderstanding of T1D and efforts to diagnose T1D more quickly. Although not carried out only through blogs but through other social media as well, a recent example of this is the advocacy efforts that led to various states adopting resolutions regarding awareness of and testing for T1D, including North Carolina (House Bill 20, so-called “Reegan’s Rule”), California (Senate Resolution 63), and Pennsylvania (House Resolution 569), for example. Such advocacy and peer-to-peer support show great potential for the utility of blogs.

However, many HCPs may be hesitant to refer patients to online blogs for support for reasons including concern about the spread of misinformation and the lack of clinical or other professional moderation of content [45]. This in-depth analysis of 140 blog posts and 663 associated comments spanning 27 months of content revealed a distinct paucity of medical misinformation. There was also a significant degree of self-moderation among blogs. Although not moderated by clinical professionals, the blog owners typically moderate comments before publishing and are acutely aware of the potential for misusing or misinterpreting information on their blogs; all contain a prominently posted statement that their content should not be considered medical advice, encouraging parents instead to obtain medical advice only from their health care team. These findings suggest that concerns about safety of blog content and lack of moderation might be unnecessarily high. Perhaps this can help HCPs reconsider how and if to add high-quality blogs to the relatively small list of support resources they can offer to T1D caregivers.

### Limitations and Strengths

One of the primary limitations of this study is one shared by virtually all research on social media: that the data are all derived from those who have chosen to express their views online, with no contribution from those who have not chosen to share. This relates to the small sample size as well; although we analyzed three blogs extensively (from among the unknowable number of T1D caregiver blogs, which likely number in the dozens or even hundreds) this is akin to conducting qualitative interviews among a small sample of individuals who represent a small fraction of the population. But balancing this are two relative strengths of the approach: (1) the inclusion of blog posts spanning a period greater than two years may allow greater depth than a typical qualitative interview of one to two hours, and (2) the inclusion of comments associated with the blog posts includes many other people in the study sample and analysis. Despite this, these findings cannot be generalized without further study, but that is an inherent and accepted characteristic of qualitative research. Another limitation is inherent to blog research itself: in contrast to more traditional qualitative methods involving personal or focus group interviews, analysis of existing blogs does not afford the opportunity to ask clarifying questions or to elaborate. The blog posts and comments must stand on their own. Although this is certainly somewhat limiting, there are significant methodological and financial benefits that are quite valuable. For example, the recruitment process is far easier, there is no scheduling involved and likewise no project costs in offering compensation to participants, and there are no transcription costs because the data are already typed by their authors. Such strengths must be balanced against the limitations of the approach. Ultimately, a combination of blog analysis and more traditional interviews may be a promising combination in approaching qualitative research. Blog analysis would reduce the time and cost associated with doing purely interview-based research, and doing some interviews in addition would afford the opportunity to pursue clarification and elaboration where that is impossible with blog analysis alone.

### Conclusions/Future Directions

This study of blogs found that caregivers to children with T1D worry about hypoglycemia, especially at night, and that the time around diagnosis is life altering and scarring, which has been found in other caregiver studies not utilizing blog analysis. These corroborations lead us to suspect that this novel research approach is able to produce valid results. Beyond that, this study provides insights into caregivers’ persistent emotions, the physical and emotional burdens they bear, benefits of incorporating newer technologies into diabetes management—and the new issues that also come with progress. Finally, blog use was found to be a vehicle for providing peer support and to allow peers to come together and encourage one another to advocate for issues they feel are important.

This study suggests that high-quality blogs can provide much-needed peer-to-peer support to caregivers of children with T1D, and other research is needed to verify that. Blogs could be considered as an adjunct to in-person support groups and as a venue for support in the many geographic areas that do not have easy accessibility to endocrinology offices [46,47]. If blog use is found more broadly to be a valid and safe means of support, practical methods and timing to incorporate this into practice will need to be established.

## Abbreviations

CGM: continuous glucose monitor
HCP: health care provider
T1D: type 1 diabetes
